# Sedentary Behavior and Physical Inactivity in the Asia-Pacific Region: Current Challenges and Emerging Concerns

**DOI:** 10.3390/ijerph19159351

**Published:** 2022-07-30

**Authors:** Michael Annear

**Affiliations:** Faculty of Sport Sciences, Waseda University, Tokyo 359-1192, Japan; annear@aoni.waseda.jp

**Keywords:** sedentary behavior, physical inactivity, Asia-Pacific, cardiovascular disease, obesity, population aging, climate change, COVID-19

## Abstract

This editorial sets the scene for our Special Issue on the growing problem of sedentary behavior and physical inactivity in the Asia-Pacific region. In many societies, more than 40% of the adult population and growing numbers of children are insufficiently physically active to safeguard their health. This is contributing to high rates of cardiovascular disease, obesity, and other deleterious health outcomes across the region. The Asia-Pacific is heterogeneous and complex, with diverse social, cultural, and environmental barriers that affect intentions and opportunities for regular physical activity. Recently, the problem has been compounded by the acceleration of population aging, the worsening effects of anthropogenic climate change, and the ongoing COVID-19 pandemic. Without strong leadership, enduring funding support, and innovative interventions that cut across policy and society, we may yet be facing a century of unmitigated expansion of morbidity across the Asia-Pacific.

## 1. Losing a Public Health Panacea

Many adults and children across the Asia-Pacific region are not sufficiently physically active to support their health [1,2]. This is surprising considering that participation in regular moderate–vigorous physical activity has a substantial evidence base showing benefits across weight management, cardiorespiratory fitness, mental health, mobility, and chronic disease prevention [3,4,5]. An equally robust body of scientific literature also attests to significant increases in morbidity and mortality associated with prolonged inactivity (a level of activity below recommended guidelines) and sedentary behavior (a state of physiological rest) [6,7]. Regular physical activity undertaken at moderate–vigorous intensity is arguably the closest thing that we have to a panacea within public health yet calls from governments of Asia-Pacific nations to Push Play [8], Move It [9], or Add 10 Minutes [10], appear to be largely unheeded at a population level.

Diverse factors are conspiring to reduce opportunity, time, and motivation for physical activity across the Asia-Pacific region. These include lack of appropriate and consistent infrastructure, inequities in access to sports facilities, poor work-life balance, changing modes of learning and leisure, COVID-19 pandemic restrictions, increasing risk aversion, and others [11,12]. At the same time, populations and environments across the Asia-Pacific are changing in ways that are likely to have major consequences for health systems and human behavior. Two key mega trends that will contribute to defining public health outcomes in the Asia-Pacific this century are population aging and anthropogenic climate change. Long-term outcomes of these social and environmental forces are difficult to predict, but they are likely to influence opportunities and intentions for physical activity among many cohorts and societies.

This Special Issue of the International Journal of Environmental Research and Public Health brings together cutting-edge research that explores a diversity of topics related to inactivity in the Asia-Pacific. The carefully curated selection includes manuscripts concerning reductions in children’s play, later life activity declines, the rise of metabolic disease risk factors, unequal distribution of behavioral impacts associated with the ongoing COVID-19 pandemic, and other thought-provoking contributions. It is hoped that this Special Issue will inform ongoing debate and stimulate new research, policy, and interventions to promote higher levels of activity across the life course in this rapidly changing region.

## 2. Pandora’s Box and the Asia-Pacific

The Asia-Pacific region has several geographical definitions, which typically comprise over 50 nations across Oceania and the Pacific, South and South-east Asia, North-east Asia, and Central Asia. This region is arguably the most populous, rapidly aging, culturally diverse, and economically disparate area of the globe [13]. This extreme heterogeneity presents challenges for addressing the growing prevalence of inactivity and sedentary behavior and developing a coordinated regional stance on the reduction of lifestyle disease risk factors and a consistent approach to educational and environmental intervention. Patterns of physical activity in everyday life are closely linked to socio-cultural norms and the vagaries of environmental opportunities and constraints that vary widely across the region [11]. Consider, for example, the diverse challenges to engaging in physical activity faced by an overworked office worker in urban Japan, an indigenous youth in outback Australia, or a single mother in Fiji. Even within national or city borders, activity opportunities and outcomes can be highly disparate, with socioeconomic status and neighborhood deprivation playing a major role in determining behaviors [14]. Consequently, understanding the nature and scale of the problem of inactivity is among the first challenges that need to be addressed by researchers and public health practitioners.

Considerable discrepancies have been reported in the measurement of physical activity across the Asia-Pacific region. This may be due to a lack of standardized measurement, which has been well documented in largescale review studies of regional data [15]. Indeed, national rates of population inactivity have been variously reported between 7 and 93%, with a median value of 38% [15]. However, when considering only research based on valid and reliable measures, such as the International Physical Activity Questionnaire (IPAQ) [16], the scale of the problem becomes clearer and more consistent. For example, national data collected in Japan among 4000 adults using a validated Japanese language version of the IPAQ showed that 40% of individuals aged 45 years and older were insufficiently physically active in a typical week [17]. Similarly, data from 4773 adult Taiwanese collected using the IPAQ showed that 42% of the sample were not sufficiently physically active to benefit their health [18]. Thus, when employing appropriate measurement tools, it appears that well over one-third of all adults in many Asia-Pacific nations are insufficiently physically active.

Mortality and morbidity data across the region also provide indirect evidence of deleterious lifestyle behaviors. For example, despite being highly preventable cardiovascular diseases (CVD) remain the leading cause of death (9.4 million deaths and over a third of total mortality) across the region [19]. Pacific Island and South-east Asian nations, including Indonesia, the Philippines, Fiji, and Papua New Guinea, have particularly high rates of CVD deaths, which fits a pattern of mortality across lower-middle income nations [20]. Rates of obesity (a precursor of life-limiting morbidity and mortality) in more developed Asia-Pacific nations are also concerningly high. For example, New Zealand and Australia are ranked as the most clinically overweight nations in the Asia-Pacific region, with 31% and 28% of their respective adult populations meeting these unenviable criteria [21]. Across the spectrum of more and less developed societies within the region, there appear to be significant preventable risk factors and disease outcomes, which are closely linked to insufficient levels of physical activity. Despite a diversity of environments and cultures, it appears that many roads are currently leading to inactive lifestyles and poor health outcomes.

## 3. Mega-Trends and a Changing Region

The regional problem of inactivity is potentially influenced by the mega-trends of population aging and anthropogenic climate change, which will define health and behavioral outcomes in many societies over the 21st century. Population aging refers to a demographic transition from a younger to an older age structure whereby growth in older adult cohorts significantly outpaces changes among cohorts of children and younger adults due to long-term reductions in fertility and increases in longevity [22]. Across the Asia-Pacific region, nations such as Japan, the Republic of Korea, Singapore, Australia, and New Zealand are at the forefront of this transition [23]. Indeed, Japan leads the global trend in population aging and is regarded as a super-aged society with >28% of its population aged 65 or older in concert with negative population growth [23,24]. While age itself is not a direct cause of morbidity, it is associated with increased risks for frailty, disability, chronic disease, and hospitalization [25,26], which creates significant pressures for health and social care. Relatedly, there is a general trend for reduced physical activity levels from 45 years of age and older, which has been well reported in the international literature [17,27]. Determinants of this phenomenon may relate to social and cultural norms regarding older adult disengagement, economic or familial pressures, detraining and sarcopenia (which reduce the capacity for activity), or a lack of appropriate environmental support for preferred activities [11].

Beyond population changes, the progression of anthropogenic climate change is also likely to affect opportunities for physical activity at a population level. Anthropogenic climate change refers to long-term changes in temperature and weather patterns linked to human industrial activities and the burning of fossil fuels [28]. The result of these changes will include more extreme weather events, including recurrent heat waves, more powerful storms, flooding, and inundation. The increased frequency and intensity of such events has the potential to limit outdoor activities in particular, including children’s informal play, school physical education, active travel, and organized sport [29]. Recently, international sports events have had to alter their programs to mitigate the effects of extreme heat linked to the progression of climate change [30]. For example, marathon and race walk events for the recent Tokyo 2020 Olympic games (held in 2021 due to the COVID-19 pandemic) were moved from the capital to the northern city of Sapporo after research findings and pre-event athlete heat injuries revealed significant risks for competitors and spectators [30]. There are also evidence-informed concerns about the ongoing viability of outdoor sport in all but the most extreme latitudes towards the end of the current century. Indeed, researchers have predicted that only eight cities outside of Europe will have climates suitable for hosting major summer sports competitions within a few decades [31]. Nations of Asia and the Pacific are likely to suffer disproportionately from the effects of climate change due to the clustering of large populations in vulnerable latitudes and coastal regions.

## 4. COVID-19 and the Amplification of Inactivity

The ongoing COVID-19 pandemic has added to the challenge of regional inactivity in several ways. Enforced lockdowns, quarantine and self-isolation, home-based and online modes of work and learning, social distancing, cancellation of sport and group exercise, and the permanent closure of businesses serving recreational physical activity needs are some of the ways in which the pandemic has altered the regular behavior of children and adults across the region. At the time of writing, the world is entering the third year of the pandemic and a seventh wave of infection with no signs of abatement despite the wide availability of relatively effective vaccines and booster shots. The historic and lingering nature of this pandemic is leading to long-term changes in physical activity patterns at a population level. This has been shown in cross-national studies using objective activity (step count) data collected from smartphone accelerometry and GPS data [32]. Such studies have shown a 27% reduction in daily population-level physical activity following the announcement of the global pandemic with a failure to return to a pre-pandemic baseline [32]. The contraction of physical activity in the Asia-Pacific region has been particularly acute, with physical activity reductions of up to 36% reported among vulnerable population groups (e.g., older Japanese) [33]. The long-term nature of the pandemic and the potential for significant detraining among vulnerable populations is arguably exacerbating the current crisis of inactivity and constraining public health efforts to promote more active lifestyles.

## 5. The Winding Path Back to Physical Activity

Persistently high levels of physical inactivity in the Asia-Pacific region portend poorly for the management of obesity, cardiovascular disease, and other deleterious health conditions in the coming decades. Population aging, climate change, and the ongoing COVID-19 pandemic are adding to the current crisis of inactivity with no near-term solutions in sight for these emerging problems. In order to tackle this complex issue, interventions at multiple levels will be required, along with strong government leadership and enduring funding commitments. International and national guidelines for physical activity have been clearly established for decades based on a robust evidence base [34], and the harms of inactivity and sedentary behavior have been equally well established [6,7]. Most national governments have guidelines for weekly or daily physical activity that align with the World Health Organization recommendations for children and adult populations [34]. What is required now is concerted and unrelenting action that cuts across policy and society in ways that create a supportive and self-reinforcing environment where activity happens naturally and often within the fabric of daily life.

Three areas for potential action that hold some promise for arresting activity declines include a renewed focus on school-based physical education, community interventions led by health professionals, and urban environmental remediation strategies. Across the Asia-Pacific, physical education programs that were once a staple of school and university life are being reduced or replaced with academic subjects at an alarming rate [35]. The value of education-based physical activity is that it can contribute meaningfully to increasing the weekly quantum of activity for younger adults while also building a skill repertoire that can support physical activity participation over the life course [35]. Novel changes to classroom learning styles with the addition of standing desks and other modes of active learning also hold some promise to increase overall activity levels in school contexts [36]. Clearly, there is a need to give school-based PE and activity interventions a stronger focus as part of educational reforms and a life course approach to healthful activity promotion in the Asia-Pacific. Beyond school-based interventions, innovative approaches are also required at the community level to address inactivity among adult populations. Public education interventions paired with health professional support may be advantageous. A good example of such an initiative is New Zealand’s Green Prescription program, a government-funded program in which community physicians provide written physical activity and lifestyle recommendations to at-risk adults and enter them into a supportive multi-week program of lifestyle change [37]. Such programs involve community support workers, nurses, and allied health professionals in a robust program of exercise and dietary change. While not universally effective, such programs have led to significant and long-term increases in physical activity for participants from high-risk populations who adhere to the program [38]. Finally, urban environments need to support and promote physical activity for a wide variety of user groups, including the very young, oldest-old, and mobility impaired. Age-friendly cities, healthy cities, barrier-free design, living streets, and urban parks movements are among a multitude of international frameworks for the creation of environments that prioritize physical activity as part of broader efforts to improve population health [39,40,41]. Community advocates and researchers are routinely engaged in auditing their neighborhood environment using valid and reliable tools in an effort to build the evidence base to drive change [42]. Irrespective of the design frameworks chosen by city planners and government officials, what is required is a long-term political consensus and sustained funding to support inclusive environmental changes that encourage physical activity such as sport, exercise, play, or transportation. The path back to activity in the Asia-Pacific region will be time and resource intensive, yet it is considerably better than the long-term expansion of morbidity that is currently confronting us.
